# Aqualogging tool for web based mapping and mitigation of soil erosion and water pollution in sprinkler irrigation systems

**DOI:** 10.1038/s41598-025-30268-y

**Published:** 2025-12-01

**Authors:** Milton José Campero-Taboada, Arturo Mampel Martín, Javier Casalí Sarasíbar, María González-Audícana, Miguel A. Campo-Bescós

**Affiliations:** 1 Departament of Engineering, IS-FOOD Institute (Innovation & Sustainable Development in Food Chain), Public University of Navarre, Campus de Arrosadia s/n, Pamplona, Navarre, 31006 Spain; 2 C.A.D.A. Ingeniería S.L. Group, Paseo Constitución Nº 25.50600 Ejea de los caballeros, Zaragoza, Spain

**Keywords:** Runoff, Green–Ampt, Erosion, Water pollution, Web mapping, Environmental impact, Civil engineering, Geomorphology, Hydrology, Agroecology, Information technology

## Abstract

This study presents a web-based tool based on the Green-Ampt model, designed to mitigate soil erosion and water pollution in sprinkler irrigation areas in Artajona, Spain. Called AquaLogging tool, its main objective is to determine the duration of waterlogging in these areas, with emphasis on soil texture to prevent surface runoff. The tool uses soil texture as a key variable to calculate water infiltration, also considering other parameters such as saturated hydraulic conductivity and initial moisture content. The methodology used simulates different irrigation scenarios to obtain different infiltration and runoff curves, which allow us to calculate the time of waterlogging for different scenarios. Through an interactive web map, AquaLogging tool allows quick access to the spatial distribution of irrigation units and soil textural classes, and with a single click we obtain detailed simulation results. In this study, three irrigation scenarios (daily, every 2 days, and every 5 days) are simulated, in which soil texture significantly influences infiltration capacity and surface runoff generation. Clay soils, characteristic of the study area, showed insufficient infiltration, resulting in runoff after short irrigation periods, while loam soils showed a higher infiltration capacity. AquaLogging tool is offered as a practical support tool for efficient irrigation management, helping farmers to make informed decisions on irrigation timing and frequency to optimise water use in irrigation.

## Introduction

Soil water erosion on cropland is a major environmental problem that triggers large soil loss^1^ and fertility loss necessitating substantial synthetic fertilizer additions for sustained productivity^2^. On irrigated land, erosion dynamics not only rainfall but also irrigation water, which can generate runoff when its application rate exceeds soil infiltration rate^3,4^.

Sprinkler irrigation, in particular, has been shown to increase runoff and erosion^5^due to the kinetic energy and high application rates of water droplets. Studies such as Santos et al^6^. report soil compaction, particle detachment and pore blockage, while Chen et al^5^. demonstrate that erosion risk depends both on soil properties and system design, underscoring the need for mitigation strategies in modern irrigated agriculture. Given its extensive and expanding use^7^, sprinkler irrigation remains a key system for improving irrigation design and management.

The movement of water on the soil surface can be influenced by land slope, which facilitates its progression. According to Morbidelli et al^8^. the influence of slope on infiltration remains uncertain due to contrasting findings between theoretical and experimental results. Gruchot et al^9^. state that changing the slope from 2.5% to 5.0% does not affect the overall surface runoff quantity but increases momentum during runoff development.

Regulatory and operational constraints, especially in Spain, highlight the need for efficient irrigation scheduling under growing water scarcity^10,11^. Increasing demands from urban and industrial sectors further restrict water availability, making it essential to employ scheduling and rotation methods to meet crop needs, reduce costs, and ensure sustainable water use.

The dynamics of runoff in irrigation systems depend largely of infiltration which controls their effects. Water absorption into soil occurs through infiltration when water reaches deeper soil layers^12^. This complex process is controlled by several climatic and soil variables^13^, which determine both the volume of water infiltrated and duration of the process^14^.

Soil infiltration determines the timing and pathway for reaching field capacity (FC)^15^ which represents the moisture content remaining in the soil after excess water is drained^16,17^. Among the variables affecting infiltration, the most relevant are soil texture, soil structure and organic matter (OM) content^18,19^. In addition, infiltration depth is an essential factor in soil water management. Several studies such as those by Hassan et al^20^., Guo^21^, Rossi et al^22^. and Cavero et al^23^. highlight its importance, as it helps to understand how water is distributed in the soil.

Infiltration models range from simple empirical to complex physics-based approaches^24,25^, each with trade-offs between simplicity, accuracy, and data requirements^24,26–28^. Physics-based models, such as the Green-Ampt (G-A) model, use fluid dynamics and soil physics principles to simulate infiltration and runoff with greater accuracy^25,29^.

The G-A model assumes a homogeneous soil profile and uniform initial moisture content^30^, and its parameters can be calculated from measurable variables such as soil texture, when experimental data are not available^31^. It is widely used to simulate infiltration^32^ and runoff processes^33,34^ due to its simplicity and adaptability, making it suitable for computational applications and practical use by farmers^13,35^. Its great flexibility allows its application in hydrological modelling, including precipitation infiltration, runoff estimation, and irrigation management^36–38^ in variable surface conditions^39^.

In response to this need, the main objective of this article is to introduce and evaluate for the first time a web-based tool called AquaLogging tool, based on the G-A model which determines waterlogging time in sprinkler-irrigated areas according to specific characteristics, aims to prevent runoff that causes soil erosion and the transport of agrochemicals due to irrigation. The need for this tool is highlighted by the growing number of farmers transitioning to sprinkler irrigation, reflecting a broader trend toward more efficient water distribution in agriculture, driven by increasing water scarcity and the need for sustainable resource management. The AquaLogging tool is intended to be accessible to farmers, managers, and designers at various stages of farm operations, including design and daily operation. Additionally, this article presents an example of the application of the AquaLogging tool in a specific location in Navarre, considering three irrigation management scenarios and local soil types.

## Materials and methods

### Study area

**Fig. 1:**
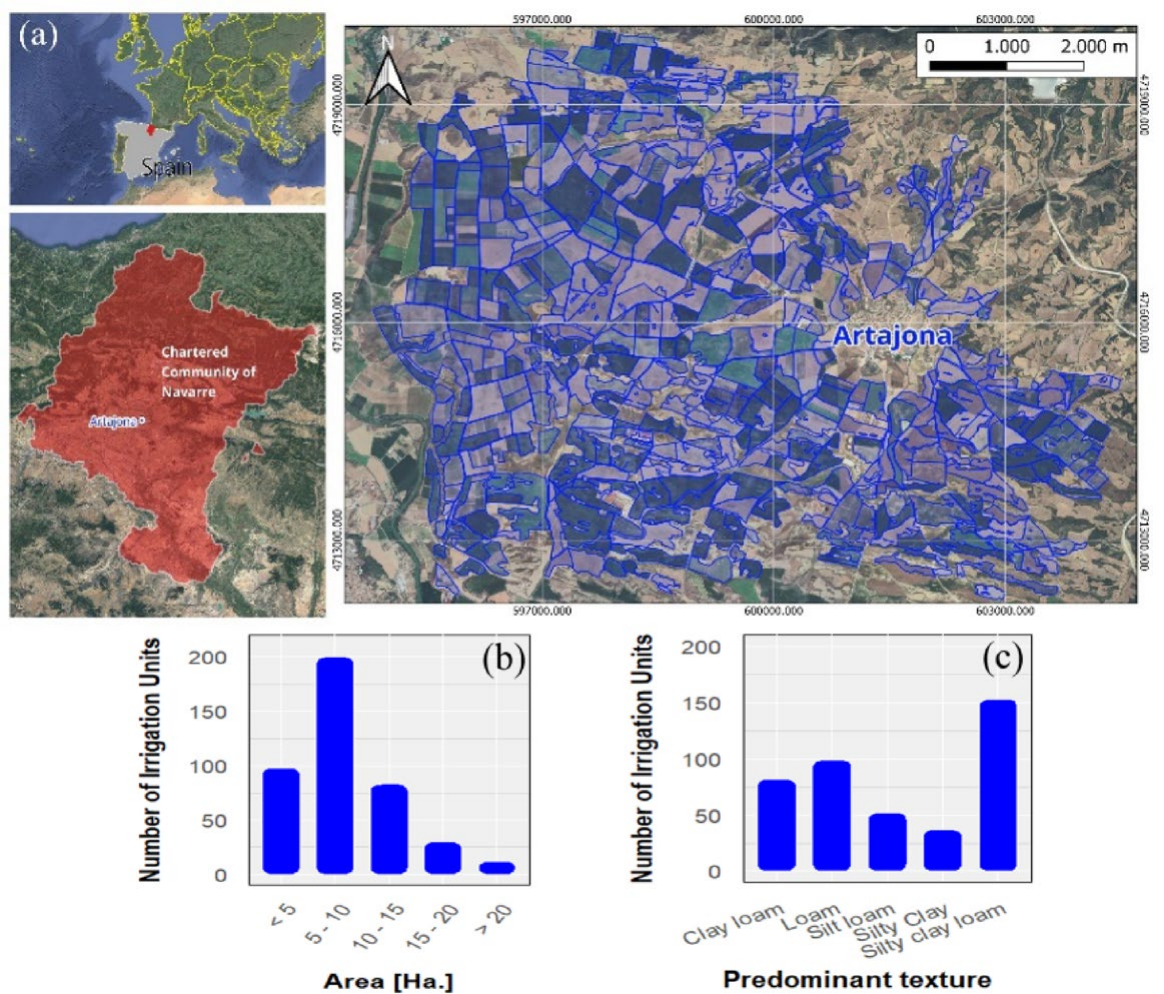
**(a)** Location map of the irrigation units in Artajona, Navarre, generated using QGIS 3.34 (https://qgis.org). Histograms at irrigation unit level of: **(b)** plot’s surface area in hectares, and **(c)** predominant soil texture created using R 4.1.1 (https://www.r-project.org).

This study was carried out in the irrigated area of the municipality of Artajona (Fig. 1a), located in the Chartered Community of Navarre, Spain. The 3534 ha. area is divided into 423 irrigation units of 1–20 ha (Fig. 1b), equipped with a sprinkler irrigation system. These plots are used to grow various agricultural crops, including the maize. Farmers adopt variable irrigation intervals, commonly implementing cycles of one, two and five days, respectively.

Within the study area, five soil texture types are identified according to USDA^40^ classification: silty clay loam, silty clay loam, loam, clay loam and silt loam (Fig. 1c). Data were extracted from the soil map of Navarre^41^. Silty clay loam is the dominant texture (153 irrigation units), while silty clay is least common (37 irrigation units).

### Green-ampt model

The infiltration process was simulated using the G-A model as part of the tool’s code, with the model’s equation^30,42^ expressed as:

1 $$\:{F}_{p}^{n+1}={K}_{s}t+M\times\:\:{S}_{av}\:\times\:ln\left[1+\frac{{F}_{p}^{n}}{M\:\times\:\:{S}_{av}}\right]$$

Where:

F_p_ = Accumulated infiltration [cm].

n = Iteration.

S_av_ = Wetting front capillary pressure head [cm].

K_s_ = Saturated hydraulic conductivity [cm/h].

t = Time [h].

M = Available porosity, calculated as $\:(\theta\:e-\theta\:i)$ [cm^3^/cm^3^].

$\:\theta\:i$ = Initial water content [cm^3^/cm^3^].

$\:\theta\:e$ = Effective porosity [cm^3^/cm^3^].

The model Eq. (1) calculates infiltration by considering constant precipitation in a homogeneous soil with a uniform initial moisture content. Soil texture is fundamental, as parameters like saturated water content, pressure head of the wetting front, and saturated hydraulic conductivity are derived from it^43^, which are obtained from Table 1, and which allowed determining the time to reach saturation and initiate runoff. The G-A model provides insights about how different types of soil affect when waterlogging starts and runoff begins through its combined assessment. It predicts the time to saturation and runoff initiation.

**Table 1:** G-A parameter values for soil textures of the study area, adapted from Rawls et al.^42^.

Texture (USDA)	Ks [cm/h]	Sav [cm]	$\:{\varvec{\theta\:}}_{\varvec{e}}\:$* [cm^3^/cm^3^]		
Clay loam	0.20016	20.88	0.279	-	0.501
Silty clay	0.10008	29.22	0.334	-	0.512
Silty clay loam	0.20016	27.3	0.347	-	0.517
Loam	1.3212	8.89	0.334	-	0.534
Silt loam	0.6804	16.68	0.394	-	0.578

^*^ *Range*.

In the context of this research, it is essential to highlight that the feasibility of determining the theoretical water infiltration depth, as indicated by Chow et al^44^., until the initiation of the runoff generation process, has been established. In this context, Eq. (2) is used to calculate the depth of water infiltrated up to the onset of runoff, based on the relationship between initial soil moisture, soil saturation and cumulative infiltration.

$$\:{\:\:\:\:\:\:\:\:\:\:\:\:\:\:\:\:\:\:\:\:\:\:\:\:\:\:\:\:\:\:\:\:\:\:\:\:\:\:\:\:\:\:\:\:\:\:\:\:\:\:\:\:\:\:\:\:\:\:\:\:\:\:\:\:\:\:\:\:\:\:\:\:\:\:\:\:\:\:\:\:\:\:\:\:\:\:\:\:\:\:\:{F}_{p}}_{\:}=L\times\:\left(\varphi\:-{\theta\:}_{i}\right)\:\:\:\:\:\:\:\:\:\:\:\:\:\:\:\:\:\:\:\:\:\:\:\:\:\:\:\:\:\:\:\:\:\:\:\:\:\:\:\:\:\:\:\:\:\:\:\:\:\:\:\:\:\:\:\:\:\:\:\:\:\:\:\:\:\:\:\:\:\:\:\:\:\:\:\:\:\:\:\:\:\:\:\:\:\:\:\:\:\:\left(2\right)\:$$

Where:

F_p_ = Accumulated infiltration until the onset of runoff [cm].

L = Depth of the water volume [cm].

$\:\varphi\:\:$= Total porosity [cm^3^/cm^3^].

$\:{\theta\:}_{i}$ = Initial moisture content [cm^3^/cm^3^].

This approach highlights the importance of understanding the interaction between infiltration and soil moisture content to accurately assess the advance of the wetting front. Furthermore, the research by Hossein Sadeghi and Troy Peters^45^, focuses on the determination of maximum irrigation depth through the consideration of key parameters such as hydraulic conductivity and maximum application rate, underlines the relevance of the topic for efficient and accurate irrigation management in agricultural environments. This parameter enhances the G-A analysis, helping farmers monitor soil water infiltration and evaluate irrigation efficiency for the entire root zone.

### Methodology for estimating waterlogging time in sprinkler-irrigated areas using the aqualogging tool

The open source software R version 4.1.1^46^. is used in this study to represent the data and make it accessible to farmers. This free open source software^47^ has packages developed by its large community, including useful functions for data manipulation^48^, irrigation^49^ and runoff^50^.

**Fig. 2:**
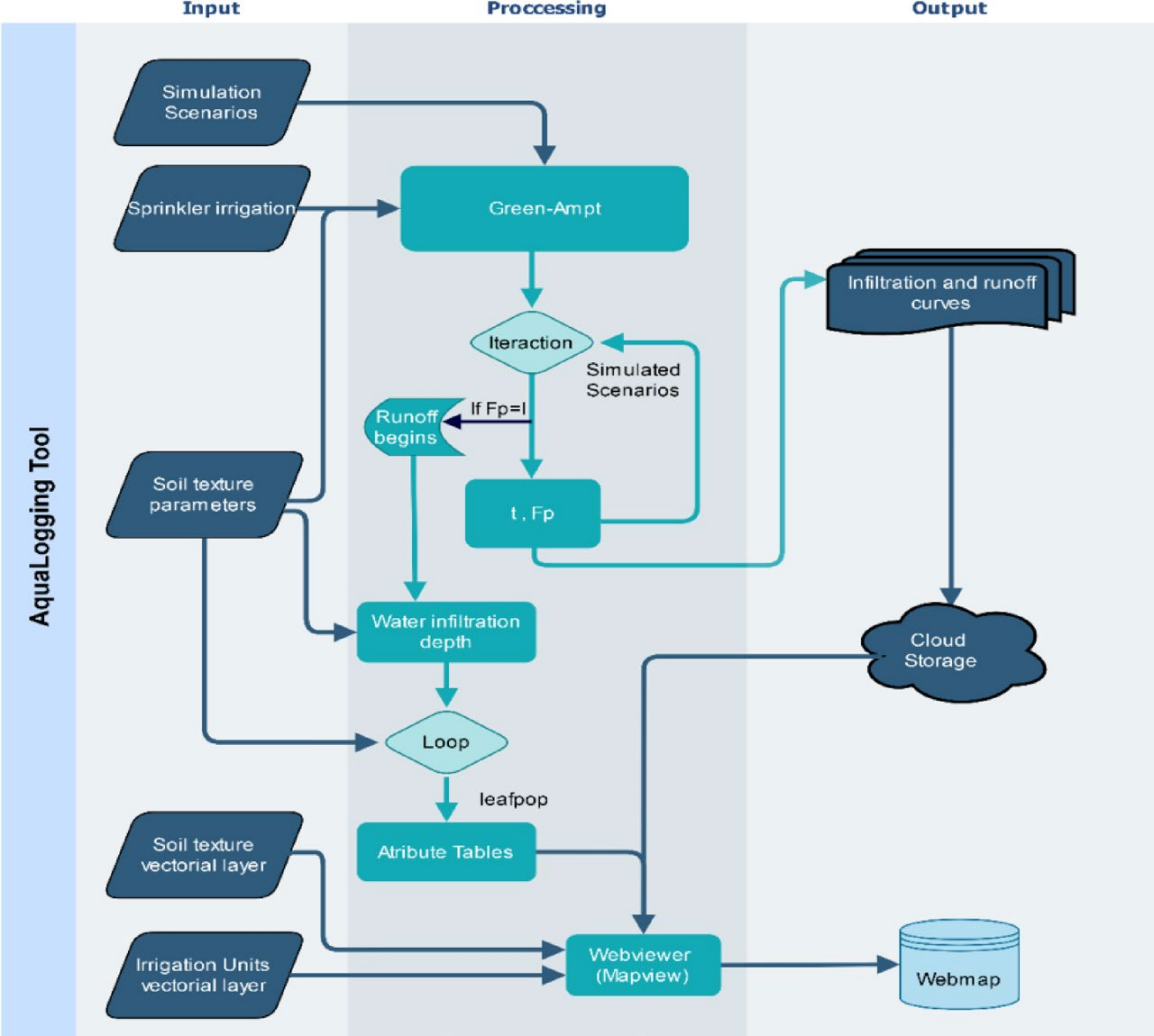
Process diagram for the generation of infiltration and runoff curves using the G-A model, estimation of the theoretical depth of water infiltration at the onset of runoff, and its presentation through a web map. The input data consider the required simulation scenarios, the amount of sprinkler irrigation water distributed, and soil texture as initial parameters. These data are processed to analyse irrigation scenarios for different time intervals, applying the G-A method iteratively to obtain infiltration and runoff curves. with the results stored on *cloud storage (Google Drive).* The web map dynamically loads images from the server when clicked, and integrates the onset of runoff and theoretical infiltration depth into an attribute table for further queries.

The AquaLogging tool is based on the G-A model, specifically adapted for application in sprinkler irrigated regions, and integrates properly with visual tools such as web maps (Fig. 2). The methodology developed starts by extracting the soil parameters required for the simulation from a csv file and the main input parameters are established in the model, including the amount of irrigation water and simulation scenarios for infiltration and runoff processes.

Simulations were performed iteratively over the different irrigation scenarios, with infiltration values calculated over time within each iteration. For each iteration, the accumulated infiltration and time data are stored, which are then used to generate the infiltration and runoff behavior curves. Waterlogging time is defined when infiltration equals irrigation intensity, indicating runoff initiation.

A matrix structure is created for simulation result data storage which includes both the waterlogging duration and irrigation advance depth measurements during different timeframes. The *ggplot* library^51^ create all-in-one images to display rainfall intensity alongside soil infiltration and runoff measurement and accumulated runoff data over time, facilitating effective analysis of the results obtained. Those rresults were exported as text files.

AquaLogging uses *Mapview* library^52^ combined with *Leafpop* library^53^ for data visualization, loading irrigation simulation data with vector layers for irrigation units and soil mapping. Leafpop generates interactive pop-ups showing our attribute tables and irrigation/runoff curves, while Mapview displays display irrigation units and soil textural classes, enhancing user interaction with the data.

### Graphic outputs of the aqualogging tool

The tool provides two types of outputs that complement each other: Infiltration and Runoff Curves, and the web map.

### The infiltration and runoff curves

**Fig. 3:**
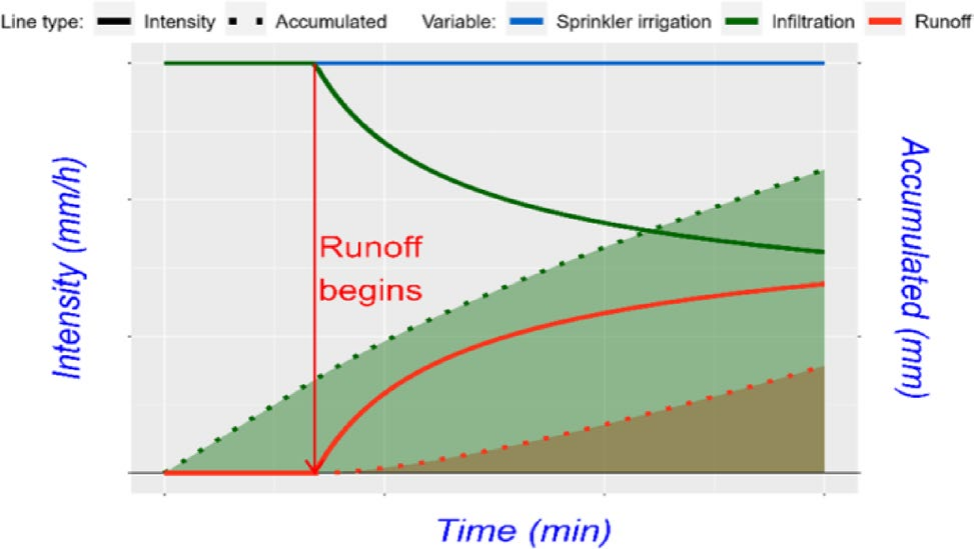
General graph of infiltration and runoff curves. The x-axis represents the time duration in minutes of the simulation scenario. The left y-axis indicates the irrigation rate in mm/h, and the right y-axis shows cumulative infiltration and runoff in mm. The solid lines represent the behavior of the variables during the simulation, blue for sprinkler irrigation, green for infiltration, and red for runoff. Dashed lines represent cumulative data, with green for infiltrated water volume and red for runoff volume. A vertical red arrow marks the point when runoff begins.

Displayed through a dual-axis graph, depict the evolution of infiltration and runoff, as illustrated in Fig. 3, where the x-axis represents the simulation duration in minutes. The left y-axis displays the irrigation amount in mm/h, while the right y-axis shows the accumulated infiltration and runoff data. The graph is divided into two sections: the first section uses solid lines to show sprinkler irrigation (blue line), infiltration (green line), and runoff (red line). The second section, with dashed lines, displays the cumulative results, with the dashed green line indicating infiltration volume and the dashed red line showing runoff and lost water. A vertical red arrow marks the point when runoff begins, ensuring clear result interpretation.

### Web map generation

**Fig. 4:**
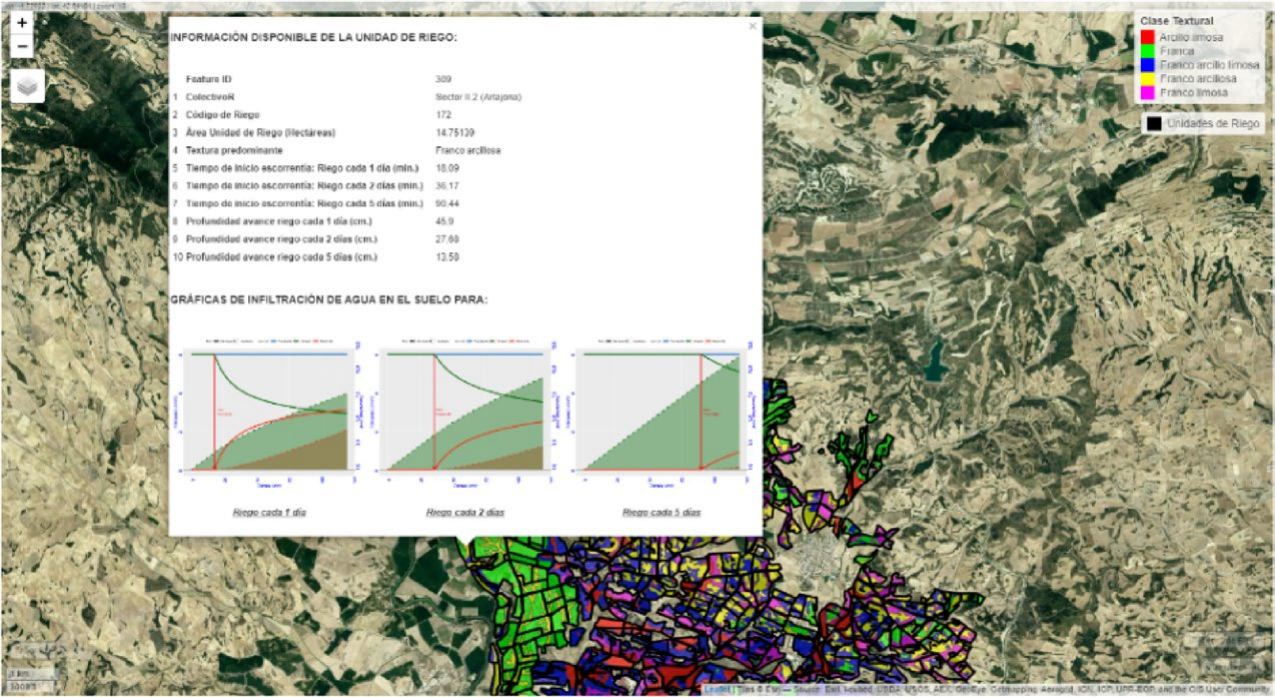
Web Map generated using R 4.1.1 (https://www.r-project.org) and the mapview package (version 2.11, https://r-spatial.github.io/mapview/). The map provides an interactive representation of spatial data, offering detailed and accessible visualization of information. It allows access to detailed tables and infiltration and runoff curves, with high-quality images downloadable from the storage cloud.

The fundamental benefit of web map (Fig. 4) technology includes an advantageous way to process and retrieve information. A user can view thorough information tables when they select an irrigation unit from the map view. During the simulation the map presents infiltration and runoff curves alongside the other sections. The curves in this system show visually the patterns of infiltration and runoff development from their temporal shifts and their relationship to other system variables. High-quality images are provided, which the system loads pictures from cloud storage. These images can be downloaded by users with a single interaction. The system uses Web Map Service (WMS) from different providers to keep base maps updated and accessible.

### Practical application of the aqualogging tool to the study area

The AquaLogging tool is implemented with initial conditions of constant sprinkler irrigation at a rate of 6 mm per hour, regardless of rainfall, with maize as the main crop. Slope was not considered in the simulations, as the Green-Ampt (G-A) model itself does not incorporate slope effects, focusing instead on soil properties and initial water content to estimate infiltration. In this study, the tool is designed to identify the exact moment when the irrigation application rate equals the soil infiltration capacity, marking the onset of runoff (Fig. 3). This simplified approach avoids dynamic flow analysis and provides farmers with a practical, rapid application of the G-A model to estimate waterlogging time under sprinkler irrigation.

Fundamental soil parameters such as effective porosity, effective suction, and hydraulic conductivity are determined based on soil texture^31,42^, with five soil types analyzed: Clay loam, Silty clay, Silty clay loam, Loam, and Silt loam. The study focuses on one of the region’s most challenging scenarios, during peak evaporation in July, to optimize irrigation for maize, which requires 6 mm of water per day during its peak demand phase^54^. In this context, we focus on analysing maize cultivation as a reference, as a practical example, due to its high water demand for development^55,56^, its widespread presence in the region, and its potential yield compared to other grains^57^.

The evaluation of runoff characteristics and maize water requirements occurs through simulations of daily, bi-daily, and five-day irrigation cycles to keep soil moisture levels at FC. The simulation spans several days to determine how the soil recovers during peak watering periods while running multiple times to study infiltration and runoff patterns. Local farming practices serve as the basis for selecting these irrigation scenarios which deliver greater precision for understanding runoff characteristics in this region. Soil texture provides information about crop water retention capacity which enables measurements of water availability in order to maintain at FC levels and reduce crop water stress and wilting conditions^58^. This approach, using maize as an example, illustrates the applicability of the tool for efficient irrigation management and sustainable agriculture.

Figure 2 depicts the G-A model process to derive infiltration and runoff curves during which the model calculates theoretical infiltration depth before runoff initiation begins. The G-A model shows runoff initiation through time-based analysis of the cumulative infiltrated water (Fp) using its mathematical Eq. (1). Successful application of this equation through an iterative process delivers specific information regarding soil texture interaction with irrigation schedules. Soil-waterlogging time depends on specific conditions particularly when irrigation surpasses the infiltration capability which leads to surface saturation. The depth of saturation zone increases until surface runoff occurs when water application exceeds a specific duration (t > tp).

Web mapping capabilities through the *Mapview* library allow geospatial data analysis while *Leafpop* library provides interactive information presentation using pop-ups along with tables and images and graphics^47,53,59^.

## Results and discussion

### Green ampt in scenario simulation: onset time of runoff

We started from the dominant soil texture data, for each of the irrigation units composing the study area (Fig. 1a). These texture data are used to define the parameters of porosity and initial water content, K_s_ and S_av_ for the G-A formula, following the approach commonly applied in other studies^24,31,32,60^.

**Fig. 5:**
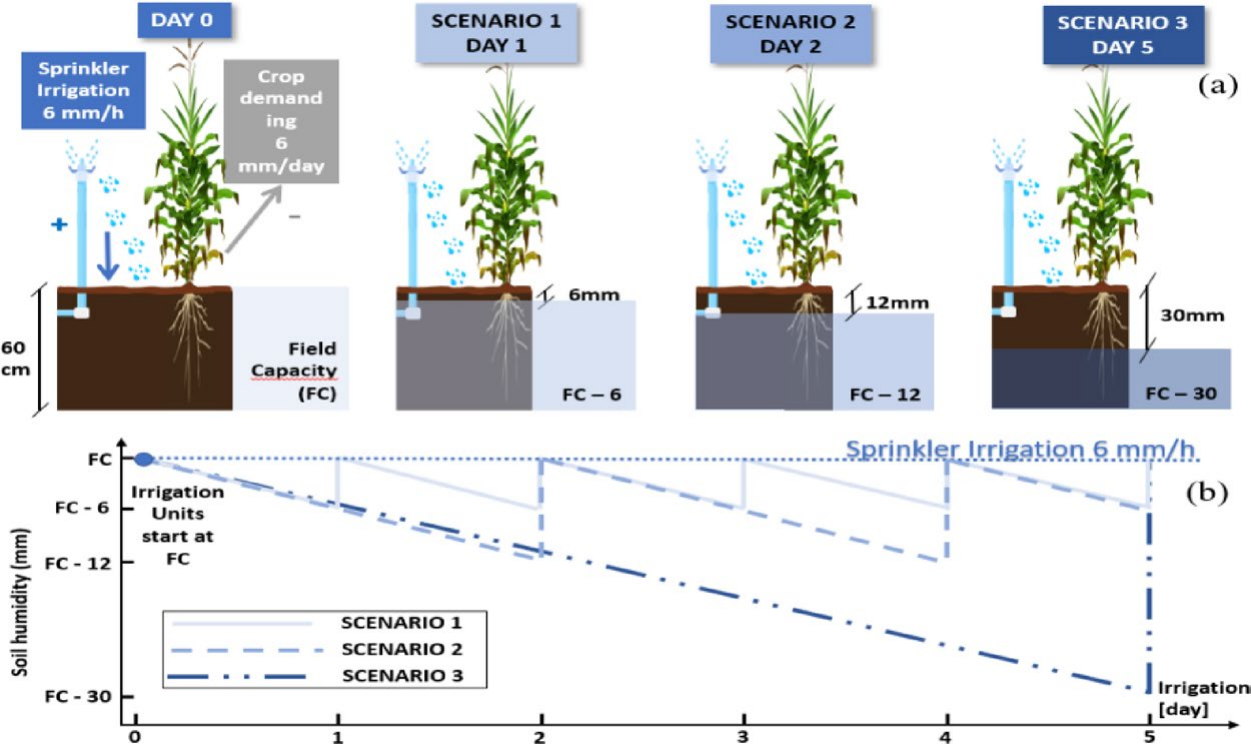
Soil moisture evolution under three irrigation scenarios simulated with the AquaLogging tool using the Green-Ampt model. Initial conditions assume soil at field capacity (FC), sprinkler irrigation with a constant application rate of 6 mm/h, and maize as the reference crop (maize crop demand of 6 mm/d). **(a)** Irrigation scheduling scenarios: daily (loss of 6 mm/day), every 2 days (12 mm), and every 5 days (30 mm). **(b)** Soil moisture dynamics for the three scenarios during a 5-day simulation period, illustrating differences in water depletion and replenishment cycles.

To determine runoff formation times, it is proposed to carry out the simulation of three irrigation scenarios (Fig. 5) with an effective depth explored by the roots of 60 cm, given that much of the water required by the plants is extracted from the top of the root zone^19^. In each scenario, soil moisture content is varied, starting from the same initial moisture content at FC in the soil profile and applying sprinkler irrigation to return the profile to its FC. With this simulation we guarantee that the roots can access to the water present in soil pores, and FC represents an optimal balance between water supply and drainage. Through the simulation of different irrigation scenarios with variations in soil moisture content based on FC, runoff formation is evaluated as a function of moisture conditions and the water requirements of the maize crop in these different scenarios.

These three irrigation intervals, corresponding to one, two, and five days respectively, are commonly used in the area and they are schematically represented in Fig. 5a and described below:

- Daily irrigation: We start with a soil wetted to FC, which loses 6 mm daily due to the water requirements of the crop (Fig. 5a: Scenario 1). Therefore, 6 mm/h of daily irrigation is applied until the water needs of the crop are met and the lost water is replenished each day. The replenishment cycle in this scenario is the shortest of the three scenarios studied, as shown in the soil moisture dynamics diagram of a daily irrigation for an irrigation simulation period of 5 days (Fig. 5b).
- Irrigation every 2 days: We start from a soil wetted to FC and, after two days, the crop loses 12 mm due to the water requirements of the crop (Fig. 5a). Consequently, irrigation of 6 mm/h is applied until the crop’s water requirements are met and the loss is replenished. In this case, irrigation demand and irrigation time are higher than in scenario 1, resulting in a higher moisture loss, as shown in the soil moisture dynamics diagram (Fig. 5b).
- Irrigation every 5 days: We start with a FC-wet soil and allow 5 days to pass, during which the crop loses 30 mm due to the water requirements of the crop (Fig. 5a). Thus, irrigation of 6 mm/h is applied, until the crop’s water requirements are met. In this case, irrigation demand and irrigation time are higher than in scenarios 1 and 2, with a higher soil moisture loss, as shown in the soil moisture dynamics diagram (Fig. 5b).

This research provides valuable insights into the temporal window when runoff begins to form, which is crucial for informed decision-making in crop irrigation management. By focusing on waterlogging time at the onset of runoff, the study justifies the exclusion of specific models, such as hydrological and soil erosion simulation models, which do not directly address this key variable. This lack of focus limits their practical utility for providing farmers with timely and relevant information to optimize irrigation timing and minimize water loss.

**Fig. 6:**
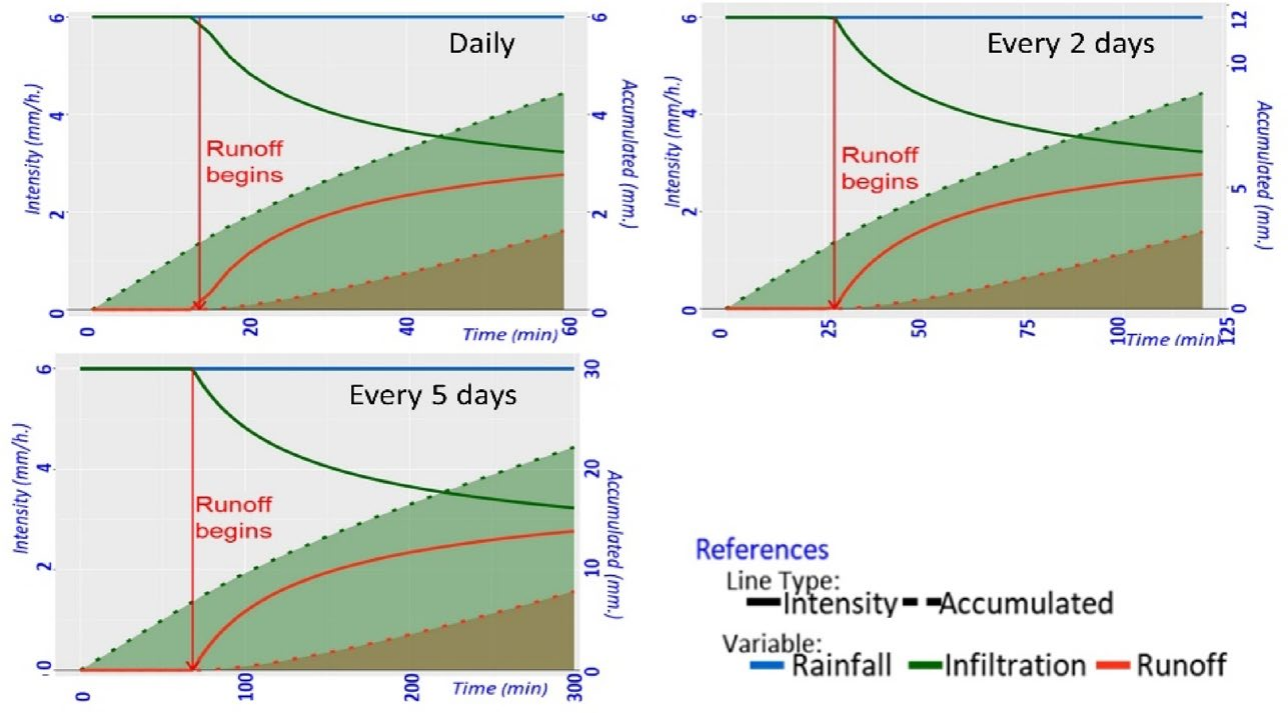
Simulation of infiltration and runoff under three irrigation scenarios: daily, every 2 days, or every 5 days, for an irrigation unit with silty clay loam texture. Initial soil moisture was set at field capacity (FC), with sprinkler irrigation applied at a constant rate of 6 mm/h. The blue line shows a constant applied precipitation, the green solid line shows the infiltration rate of the studied soil, the red solid line represents the runoff rate, the difference between precipitation and infiltration. Dotted lines show the cumulative values of infiltration (green) and runoff (red)and finally, the shaded areas under the cumulative curves indicate the accumulation of infiltrated water (green) and runoff (red).

The study area includes 36% of its total land area with silty clay loam texture in the irrigation units (Fig. 1). The infiltration capacity of the soil proved to be inadequate to stop water accumulation and waterlogging occurrence in all three irrigation scenario tests. The process of runoff formation started after 13.67 min in one scenario and 27.33 min in another scenario while the third scenario took 68.33 min. The water volume accumulated in the soil area did not surpass its natural infiltration capacity according to Fig. 6.

The study demonstrates that initial soil moisture plays a central part in runoff formation while illustrating how changes in irrigation timing affect system operational efficiency. These aspects are fundamental for a more effective water management. Therefore, it is advisable that, irrespective of the soil type, the runoff start time is not exceeded during irrigation. It is suggested to apply the irrigation rate in irrigation cycles equal to or below the estimated run-off time to avoid water loss and soil erosion in irrigation units. Another aspect to consider is to change the sprinkler frame or use sprinklers with a lower working pressure to reduce the rainfall intensity due to irrigation.

**Fig. 7:**
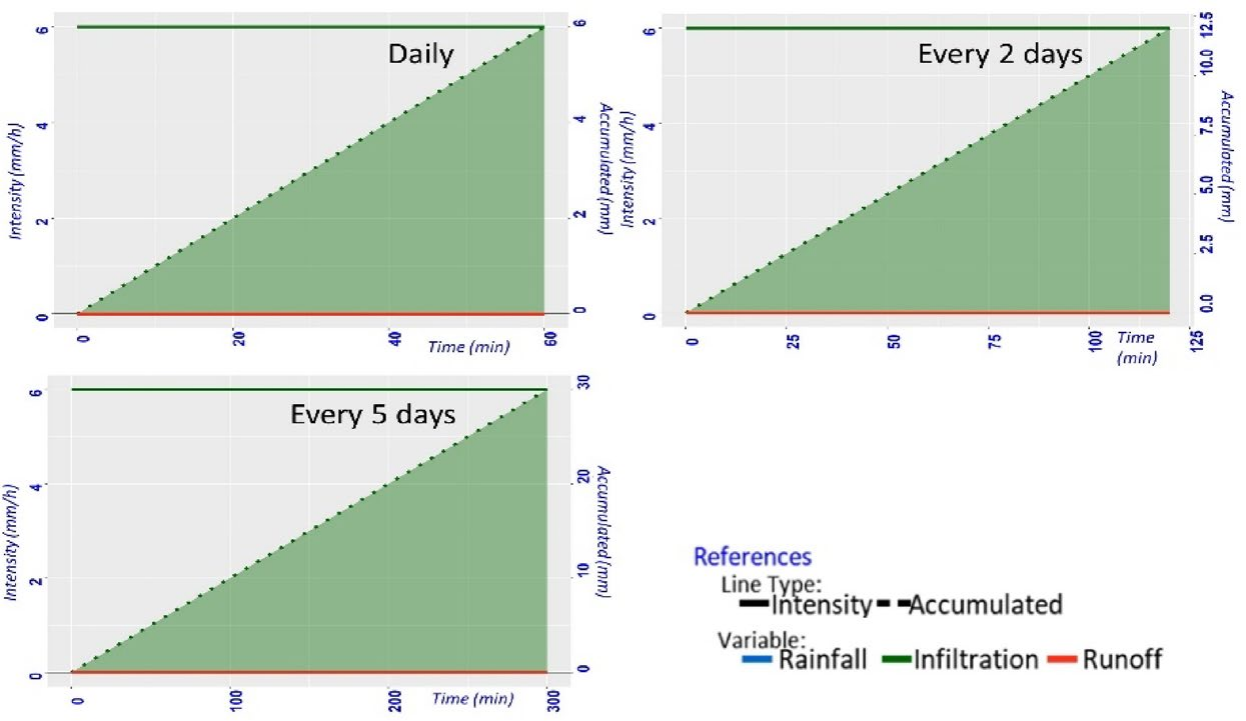
Simulation of infiltration and runoff during a two-hour irrigation period for a loam-textured irrigation unit. Initial soil moisture was set at field capacity (FC), with irrigation applied at a constant rate of 6 mm/h. Due to the high hydraulic conductivity of loam soil, no runoff was generated in any of the three irrigation scenarios (daily, every 2 days, and every 5 days); therefore, the graphs for all scenarios are identical, showing only the cumulative infiltration curve (green-dotted line) and the shaded green area under the curve indicates the volume of water infiltrated.

On the other hand, loam-textured soil (23% of the study area) does not exhibit runoff in any irrigation scenario (Fig. 7). The high hydraulic conductivity of this soil type (Table 1) enables total penetration of irrigation water which is visualized through its straight cumulative infiltration line (Fig. 7). This offers flexibility for farmers with loam-dominated plots. However, it is important to note that loam soils are prone to surface crusting^61^, which increases the risk of waterlogging.

The average runoff onset time for the daily irrigation scenario was 11.6 ± 2.7 min, increasing to 23.3 ± 5.4 min for 2 days and 58.2 ± 13.5 min for 5 days. The minimum and maximum runoff onset values ranged from 5.9 to 68.3 min across all simulated scenarios. These results indicated a clear increase in runoff onset time as irrigation intervals lengthened, reflecting the cumulative infiltration potential of the soil. Moderate standard deviations suggest relatively consistent responses within each irrigation scenario, supporting the reliability of the G-A model results.

The results obtained in this research confirm the direct influence that soil texture has on the infiltration capacity and surface runoff generation, as documented by previous studies^62–66^, however, this research validates this relationship in a simulation context, using irrigation hours specifically used by farmers, allowing a realistic approximation to field irrigation conditions.

The G-A model applied in this study relies on average parameter values derived from soil texture classes, which provide a practical basis for estimating infiltration and waterlogging times in sprinkler-irrigated areas^60,67^. This approach has the advantage of being broadly applicable, as highlighted by Rawls et al^42^. and Deng and Zhu^60^, even though local soil management practices, tillage, and structural variations can substantially modify infiltration behavior. Therefore, while the use of recommended texture-based averages ensures general validity, it does not fully capture site-specific variability. Future research could address this limitation by incorporating field measurements to refine the model and enhance its accuracy for specific locations as shown in studies conducted by Beitlerová et al^68^.

In terms of accuracy, the runoff onset times estimated using the Green-Ampt model for clay loam are consistent with this texture’s susceptibility to surface sealing and its low final infiltration capacity^13,69^. The rapid onset of runoff is consistent with studies showing reductions in infiltration in silty loam soils after several irrigation events^70^. Conversely, the absence of runoff in loamy soils coincides with their higher infiltration rates described in the literature^69^, confirming the validity of the results obtained.

However, infiltration is strongly influenced by local factors such as soil structure, crust formation and management practices^70^. Previous studies have shown that differences in measurement methods can alter infiltration rates by up to an order of magnitude^71^, and that the presence of macro-soil pores or crop residues can significantly alter water dynamics^13^. In this regard, future studies could consider future validation with field data to capture the spatial and structural variability of the soil and refine the accuracy of the model.

The theoretical water infiltration depths (L) up to runoff onset vary based on soil textures and irrigation frequency, including daily, every 2 days, and every 5 days (Table 2). This analysis is made under the same original simulation conditions include soils initially wetted to FC and the application of sprinkler irrigation.

The research findings match those presented by Hossein Sadeghi and Troy Peters^45^ regarding the variation of irrigation breakthrough depths according to soil textures and irrigation frequency. Soils with higher infiltration capacity enable deeper water distribution, promoting root access to moisture and nutrients. Soil texture strongly influences infiltration parameters like hydraulic conductivity. This information is valuable for farmers, as it helps determine how much irrigation water reaches crop roots and supports efficient water management.

The irrigation strategy in the simulation restores soil moisture to FC. A constant irrigation rate of 6 mm/h was maintained, while the daily evapotranspiration rate was also considered constant. Adjustments were made for irrigation frequencies of daily, every 2 days, and every 5 days (Fig. 5a). Despite differences in irrigation duration infiltration depths remained similar for the same soil type (Table 2). This strategy promotes uniform water distribution, aiding root development and crop growth. Irrigation frequency influences both infiltration depth and soil moisture replenishment.

**Table 2:** Irrigation advance depth (L).

Texture	L 1 day (cm)	L 2 days (cm)	L 5 days (cm)
Silty clay	30.37	30.37	30.37
Loam	60.00	60.00	60.00
Silty clay loam	44.40	44.40	44.40
Clay loam	41.29	41.29	41.29
Silt loam	60.00	60.00	60.00

### Visualization of agricultural data: sharing data on web map

The web map developed using R code (Fig. 4) has been constructed using the *mapview* and *leafpop* libraries, based on two main vector layers: one focused on the distribution of irrigation units and another highlighting soil textural classes. Both layers offer informative pop-ups that provide specific details and direct links to images presenting the results of the infiltration and runoff simulation in three irrigation events. These images are related to each geographical entity, showing the results of the simulation for daily, every 2 days, and every 5 days irrigation cycles. The interface provides context-based navigation for users and allows easy integration of new data layers (Fig. 4). The web map supports farmers in crop analysis by examining multiple soil variables and identifying production hotspots in line with recommendations from previous studies^72^.

The AquaLogging tool was developed using open-access data, minimizing privacy concerns and simplifying deployment. This aligns with platforms such as DAKIS^73^, where interoperable structures facilitate adoption while maintaining ethical standards. In contrast, Zipper et al^74^. caution that high-resolution spatial or socio-economic data can pose privacy risks; however, AquaLogging relies exclusively on non-sensitive, aggregated datasets. Additionally, web map programming integrates WMS services (e.g., Esri, OpenStreetMap) to ensure seamless interoperability^75,76^.

The tool is scalable allowing new irrigation units, soil types, or simulation results to be added without modifying the core architecture, and it supports role-based access for different users, ensuring both practicality and relevance. Furthermore, the architecture allows for potential future integration with IoT systems and real-time data processing^77^, enabling dynamic calculations directly in the field, although the current approach remains conceptual and simulation-based.

## Conclusions

Modern irrigation faces continuous obstacles that need innovative solutions in order to meet current requirements. Sprinkler irrigation creates a need to minimize the production of runoff which endangers agricultural soil and moves agrochemicals along irrigation systems. The rising food requirements alongside expanding irrigation zones require effective practices for irrigation management. This article presents introduction details and early evaluation of AquaLogging tool which enables farmers along with designers to control irrigation systems under current irrigation conditions. Using AquaLogging one can estimate waterlogging durations at parcel level based on soil parameters and sprinkler irrigation along with water demands and irrigation strategies. It represents a critical digital tool for modern agriculture and can play an important role for achieving rising production goals with sustainable practices.

The AquaLogging tool demonstrates its potential to estimate waterlogging durations accurately through the examined practical circumstances that involve diverse irrigation approaches. As a tool which integrates soil information with irrigation requirements it enables users to make informed decisions about how to schedule and distribute irrigation sessions. The detailed irrigation management system performs critical roles in optimizing water usage efficiency alongside flood protection measures for the environment.

The implementation of AquaLogging as part of agricultural practices contributes to the ongoing agricultural digitization movement. The future advancement of technology will deepen the utility of tools like AquaLogging for agricultural resource optimization and sustainability improvement.

Additionally, the practical example of the AquaLogging tool, based on the G-A model, highlights the significant impact of soil texture on runoff generation and its implications for crop cultivation. Predominantly clayey soils (covering 36% of the study area) demonstrated insufficient infiltration capacity, resulting in runoff during short irrigation periods, while loamy soils (covering 24% of the study area) exhibited higher infiltration capacity, allowing for more adaptable irrigation practices. However, surface crust formation in loamy soils becomes a pertinent consideration, which can be mitigated by employing cycle irrigation strategies.

Observations revealed that soil characteristics significantly influence water distribution within the soil profile. Soils with tend to distribute water more effectively to greater depths. Conversely, high clay content, coupled with reduced irrigation frequency, may limit water infiltration into deeper soil layers, potentially hampering root development in crops such as maize.

Moreover, the generated web map tool included as a supportive resource in AquaLogging tool, enables interactive visualization of irrigation unit distribution, soil textural classes, and other relevant parameters. Detailed information, including runoff times and calculated irrigation depths, is available by clicking on specific irrigation units. The integration of images resulting from infiltration and runoff curves enhances the practical utility of the web viewer, enabling farmers to assess the effectiveness of their irrigation practices and adjust as needed to minimize water loss and reduce environmental impact.

Aspects for future improvement include enhancing the web map tool for greater intuitiveness, incorporating advanced visualisation and analysis features, allowing customisation to meet specific user needs, and exploring tool expansion to cover a broader range of crops and environmental conditions. It is important to note that this summer example does not necessarily represent the most unfavourable scenario. Other scenarios, such as spring irrigation with high moisture content, could also be critical and should be considered in future research. These improvements aim to optimise the tool’s usefulness and versatility, benefiting farmers and agricultural professionals in the efficient and sustainable management of irrigation.

## Data Availability

The datasets used and/or analysed during the current study available from the corresponding author on reasonable request.
